# User Engagement With and Perceived Impact of a Digital Cognitive Training App on Cognition, Daily Functioning, and Mental Fitness: Secondary Analysis of Cross-Sectional Survey Data

**DOI:** 10.2196/80027

**Published:** 2025-10-10

**Authors:** Kelsey McAlister, Lara Baez, Anna Braunsdorf, Yana Yang, Dan Kessler, Jennifer Huberty

**Affiliations:** 1 Fit Minded, Inc Phoenix, AZ United States; 2 The Mind Company San Francisco, CA United States

**Keywords:** cognitive functioning, mobile health, real-world evidence, digital health, app-based intervention, mental performance

## Abstract

**Background:**

Cognitive difficulties are common and can interfere with daily functioning. While digital cognitive training apps are widely used, few studies have examined whether personalized tools support perceived improvements in cognitive functioning, daily functioning, and overall mental fitness among general adult users.

**Objective:**

The purpose of this secondary analysis was to explore the self-reported cognitive benefits of Elevate, a commercial, personalized cognitive training app developed to support cognitive functioning, as well as engagement with the app. We aimed to (1) describe demographics, engagement metrics, and self-reported improvements; (2) examine associations between app engagement and self-reported improvements in cognitive functioning skills directly targeted by the app; and (3) examine associations between app engagement and self-reported improvements in daily functioning and overall mental fitness as potential transfer effects of cognitive training.

**Methods:**

Adult Elevate users (aged ≥18 years) who used the app at least twice in the previous 30 days completed a brief web-based survey on perceived cognitive, functional, and mental fitness improvements. Responses were linked to objective app use data, including total active weeks, mean active days per week, and mean time per day. Ordinal logistic regressions tested associations between engagement metrics and self-reported outcomes controlling for demographic variables. A Bonferroni correction was applied to adjust for multiple comparisons.

**Results:**

A total of 3367 adult Elevate users were included. Participants were primarily middle-aged (mean 55, SD 16 y), White (2557/3336, 76.65%), and female (2184/3362, 64.96%), with 67.72% (2274/3358) holding at least a college degree. Using the app across more weeks was associated with a greater likelihood of reporting improvements in all cognitive skills (odds ratios [ORs] 1.0014-1.0027, 95% CI 1.0006-1.0036), several areas of daily functioning (eg, motivation and task efficiency; ORs 1.0014-1.0017, 95% CI 1.0006-1.0026), and overall mental fitness (OR 1.0021, 95% CI 1.0012-1.0031). More days of use per week were linked to improvement in math only (OR 1.15, 95% CI 1.09-1.22), whereas spending more time per day was associated with improvements in speaking, reading, math, motivation, personal progress, and mental fitness (ORs 1.02-1.04, 95% CI 1.01-1.06).

**Conclusions:**

Greater use of the Elevate app was linked to self-reported improvements in cognitive skills, daily functioning, and overall mental fitness. These findings suggest that personalized, adaptive cognitive training apps such as Elevate may serve as scalable tools for enhancing everyday cognitive and functional well-being. Future research should use rigorous, longitudinal methods to confirm these effects and clarify which app features drive meaningful outcomes.

## Introduction

### Background

Cognitive difficulties such as challenges with memory, attention, and information processing are increasingly common, with estimates suggesting that 2 out of 3 Americans will experience some level of cognitive difficulty [1]. Even minor declines in cognitive abilities (eg, slower processing and reduced working memory) can impair everyday tasks such as managing finances and organizing schedules [2-4]. When these difficulties persist over time, they are associated with more than twice the risk of developing mild cognitive impairment or dementia within the next decade [5]. This risk increases as individuals age as 75% of older adults with ongoing cognitive concerns go on to develop clinical symptoms within 10 years [5]. These everyday difficulties are often intensified by stressors such as chronic stress, frequent multitasking, and digital overload, which place ongoing strain on attention and executive function [6,7]. For example, multitasking between multiple forms of screen media (eg, texting, watching television, and engaging with social media) has been associated with greater cognitive difficulties, including poorer working memory, reduced attentional filtering, and increased vulnerability to distraction [7]. As such, there is a growing need for scalable interventions that prevent cognitive difficulties and promote cognitive functioning in everyday life.

Mental fitness is a novel framework for assessing cognitive difficulties. Although it is an emerging concept that has not yet been empirically defined, we define mental fitness as a state of optimal cognitive and emotional functioning, supporting the ability to regulate emotions, think clearly, and apply knowledge effectively to navigate daily demands [8-13]. Rather than focusing solely on disease prevention or symptom reduction, mental fitness emphasizes the ongoing development of capacities that support real-world cognitive functioning and adaptability [1,11,13]. While related to constructs such as cognitive health and executive function, mental fitness is a dynamic, trainable construct that integrates both cognitive and emotional domains and is theorized to support everyday functioning. While mental fitness is gaining traction as an important construct, it lacks both a universally accepted definition and valid measurement approaches [8-11]. Nonetheless, this novel framing offers a useful theoretical foundation for evaluating how tools and practices may enhance cognitive functioning and prevent decline, especially in nonclinical or wellness-oriented populations.

A variety of interventions have been developed to prevent cognitive difficulties and support cognitive functioning as a core component of mental fitness, including lifestyle interventions (eg, physical activity, mindfulness, and stress management) and compensatory strategies (eg, note-taking and time management techniques) [14-16]. Lifestyle interventions may promote general brain health but typically do not involve targeted practice of specific cognitive skills [15,16]. Compensatory strategies help individuals work around cognitive difficulties but also do not target underlying cognitive abilities [14]. In contrast, cognitive training (ie, the active use and challenge of mental skills) is a promising pathway for maintaining or enhancing cognitive function, with evidence linking intellectually stimulating activities to lower risk of cognitive difficulties and clinical impairments (eg, dementia) [17-19].

A growing number of digital cognitive training tools aim to address cognitive difficulties and support functioning through brief exercises, often delivered via mobile apps targeting aspects of cognitive functioning such as attention, memory, and processing speed. While studies of research-based and commercial platforms have shown improvements in cognitive performance on specific trained tasks, research linking app-based cognitive training to related untrained tasks (ie, near-transfer effects) and real-world functioning (ie, far-transfer effects) is mixed [20,21]. Meta-analyses have shown that cognitive training tools may lead to improvements beyond the specific tasks trained. However, it remains unclear whether these gains translate into perceived improvements in real-world functioning or broader aspects of mental fitness, particularly among users outside of controlled research settings [20,22,23]. Furthermore, many studies evaluating the effectiveness of digital tools for cognitive decline have been conducted in specialized populations (eg, older adults and individuals with cognitive impairments), limiting generalizability to the broader adult population [24-27]. Although several commercial platforms have demonstrated effectiveness in real-world users [28-31], few studies have evaluated apps with more advanced technological features, such as a wide range of content coverage and adaptive personalization. These gaps point to the need for more research on commercial apps that incorporate more advanced features to determine their potential to support cognitive functioning in everyday life and overall mental fitness.

### Objectives

The purpose of this secondary analysis was to explore app engagement and the self-reported cognitive benefits of Elevate, a commercial, personalized cognitive training app developed to support cognitive functioning. We aimed to (1) describe demographics, engagement metrics, and self-reported improvements; (2) examine associations between app engagement and self-reported improvements in cognitive functioning skills directly targeted by the app; and (3) examine associations between app engagement and self-reported improvements in daily functioning and overall mental fitness as potential transfer effects of cognitive training. This study offers novel insights by evaluating a personalized commercial cognitive training app in a real-world context, linking objective use data with self-reported outcomes, and exploring whether cognitive training supports broader mental fitness beyond task-specific performance. The findings will help clarify the potential of widely accessible platforms to serve as scalable tools for promoting everyday cognitive health in general adult populations.

## Methods

### Study Design and Participants

This study was a cross-sectional observational analysis involving secondary use of survey and app use data. Participants were adult users (aged ≥18 years) of Elevate, a cognitive training app, who met the following inclusion criteria: (1) ability to read and understand English, (2) being located within the United States, and (3) engagement with the app at least twice in the previous 30 days. Engagement was defined as completing any activity within the app, such as a game-based training exercise. Eligible users were identified via back-end data and invited via email (sent up to 2 times) to complete an optional survey assessing their perceptions of the Elevate app’s impact on cognitive skills and everyday functioning. Participants who completed the survey were entered into a drawing to win a US $50 gift card; this incentive was offered by the company and was independent of the research protocol. The survey was administered between June 10 and 23, 2025. User engagement data were extracted from Elevate’s back-end activity logs, spanning April 18, 2014, to June 23, 2025, and were linked to survey respondents to support exploratory analyses of perceived outcomes in relation to app engagement. Among 84,505 eligible users invited to participate via email, 4190 (4.96%) opened the survey, and 3367 (3.98%) completed it in full. This yielded an 80.36% (3367/4190) completion rate among users who accessed the survey, which, in addition to the response rate, is consistent with response patterns observed in large-scale, voluntary online surveys and still supports valid inferences given the sample size and analytic approach [32-34]. Only completed surveys were included in the analytic sample.

### Ethical Considerations

The study protocol was reviewed by the Biomedical Research Alliance of New York Institutional Review Board (25-149-1708) and determined to be exempt under US federal guidelines for research involving human participants. Because the study met criteria for exemption, informed consent was not required. All data were deidentified before analysis to protect participant confidentiality.

### Cognitive Training App

Elevate is a commercially available mobile app designed to enhance cognitive skills through personalized, curriculum-based training. Users find Elevate via app store searches (eg, Apple App Store and Google Play Store), digital advertisements, word of mouth, and media coverage. The app was developed by The Mind Company, with content created by an internal team of curriculum designers, cognitive scientists, and subject matter experts. Drawing on principles from cognitive science, Elevate delivers short, interactive exercises intended to strengthen real-world cognitive functioning rather than isolated task performance.

Training is organized across 5 primary skill domains: writing, speaking, reading, math, and memory. Writing modules target clarity, grammar, and vocabulary to improve written communication. Speaking modules focus on diction, pronunciation, and word retrieval to support clear and confident verbal expression. Reading modules address comprehension, processing speed, and vocabulary development. Math modules emphasize practical numeracy skills, including estimation, proportions, and financial calculations. Memory modules include tasks designed to improve short-term recall, sequencing, and synthesis. An adaptive learning algorithm adjusts difficulty based on user performance, enabling tailored progression. Immediate feedback is provided, and users can track their progress and compare their performance to peer benchmarks.

### Self-Reported Improvements in Cognition, Daily Functioning, and Mental Fitness (Outcomes)

Survey questions were created specifically for this study and were not taken from existing standardized tools. The survey was developed by 2 doctoral-level researchers with expertise in behavior change, complementary health approaches, and digital health. The survey was designed to evaluate users’ perceptions of how using the Elevate app affected their cognitive skills, daily functioning, and overall mental fitness. These topics were selected because they closely aligned with the app’s training goals and the anticipated real-world benefits of cognitive training. The survey was web based (delivered using SurveyMonkey [SurveyMonkey Inc]) and took most participants 5 to 10 minutes to complete. All responses were anonymous. Participants completed 27 quantitative items assessing (1) perceived changes in specific cognitive skills targeted by the app (14 items) and (2) perceived changes in overall mental fitness and daily functioning since using Elevate (13 items). All items were rated on a 5-point Likert scale ranging from “Much worse” to “Much better.” At the end of the survey, participants completed demographic questions, including age, gender, race and ethnicity, educational level, English-language fluency, and employment status. Although the survey included additional items, only a subset of items relevant to the aims of this study was analyzed and is reported in this paper. Table 1 summarizes the cognitive skills, daily functioning, and mental fitness domains examined in this study and the specific survey items used to assess each domain.

**Table 1:** Cognitive skills, daily functioning, and mental fitness domains and their corresponding survey items.

Domain			Survey item
**Cognitive skills**			
	Writing	“Writing clearly and effectively”	
	Speaking	“Speaking clearly and confidently”	
	Reading	“Understanding what I read”	
	Math	“Doing mental math quickly and accurately”	
	Memory	“Remembering things when I need to”	
**Daily functioning**			
	Task efficiency	“Completing tasks efficiently”	
	Spending time intentionally	“Spending my time in ways that feel intentional and worthwhile”	
	Keeping up with responsibilities	“Keeping up with personal and work responsibilities”	
	Feeling motivated	“Feeling motivated to grow or improve”	
	Making personal progress	“Feeling a sense of making personal progress”	
**Overall mental fitness**			
	Mental fitness	“Overall mental fitness (ie, how sharp, calm, and knowledgeable you feel)”	

### App Engagement Data

Objective engagement data were obtained from Elevate’s internal use tracking system starting from each user’s first recorded activity and linked to survey responses. We examined 3 app engagement metrics. Total active weeks was defined as the number of weeks in which a user engaged with the app at least once. Mean active days per week was calculated as the average number of days in which users used the app at least once during each active week. Mean time per day (in minutes) was defined as the average duration of app use on active days.

### Statistical Analysis

Descriptive statistics, including mean, SD, total counts, and the corresponding percentages, were calculated for demographic variables, app engagement variables, and self-reported improvement variables. A series of ordinal logistic regressions (cumulative link models [35]) were used to test the effects of the number of total active weeks, mean active days per week, and mean time per day on self-reported improvements in cognitive skills (writing, speaking, reading, math, and memory), daily functioning (task efficiency, spending time intentionally, keeping up with responsibilities, feeling motivated, and making personal progress), and overall mental fitness. The proportional odds assumption was tested for predictors of interest in each model (Multimedia Appendix 1). Each ordinal dependent variable was modeled separately. All models included the effects of covariates, including age, gender, race, ethnicity, educational level, and time since downloading Elevate, to reduce confounding. All predictors were retained in all models regardless of statistical significance to maintain comparability across analyses. All models were conducted on complete cases (ie, no missing predictor, outcome, or covariate data). Odds ratios (ORs) were calculated from the model estimates. To adjust for multiple comparisons across 11 ordinal regression models with 3 main predictors, a Bonferroni correction was applied. The α level was adjusted to α=.05/33=.0015, and statistical significance was evaluated at the corrected threshold [36]. All analyses were conducted in R (version 4.3.1; R Foundation for Statistical Computing) [37]. This study is reported in accordance with the STROBE (Strengthening the Reporting of Observational Studies in Epidemiology) guidelines, with the completed checklist provided in Multimedia Appendix 2.

## Results

### Descriptive Statistics of Demographic Variables

Users (N=3367) were, on average, aged 55 (SD 16) years, mostly White or European (2557/3336, 76.65%), non-Hispanic (3113/3341, 93.18%), and native English speakers (3063/3360, 91.16%), and most identified as women (2184/3362, 64.96%). Users were also highly educated (2274/3358, 67.72% had at least a bachelor’s degree) and mostly employed (1481/3359, 44.09%) or retired (1153/3359, 34.33%; Table 2).

**Table 2:** Demographic characteristics of Elevate users.

Characteristic		Values
Age (y), mean (SD)		55 (16)
**Race (n=3336), n (%)**		
	American Indian or Alaska Native	17 (0.5)
	Asian or Asian American	202 (6.1)
	Black or African American	266 (8)
	MENA^a^	12 (0.4)
	Multiracial	194 (5.8)
	Native Hawaiian or Pacific Islander	5 (0.1)
	White or European American	2557 (76.6)
	Other	83 (2.5)
**Gender (n=3362), n (%)**		
	Woman	2184 (65)
	Man	1105 (32.9)
	Gender diverse	29 (0.9)
	Other	10 (0.3)
	Declined to state	34 (1)
Hispanic ethnicity (n=3341), n (%)		254 (7.6)
**English fluency (n=3360), n (%)**		
	“I am a native speaker of English”	3063 (91.2)
	“I speak English fluently” (near native)	213 (6.3)
	“I speak English well” (intermediate)	79 (2.4)
	“I speak a little English” (basic or novice)	3 (0.1)
	“I do not speak English”	2 (0.1)
**Educational level (n=3358), n (%)**		
	11th grade or lower	9 (0.3)
	High school diploma or GED^b^	183 (5.4)
	Some college	465 (13.8)
	2-y college or technical school degree	379 (11.3)
	Bachelor’s degree or equivalent (eg, BA or BS)	1186 (35.3)
	Graduate degree or equivalent (eg, MA, MD, or PhD)	1088 (32.4)
	Other	48 (1.4)
**Employment status (n=3359), n (%)**		
	Full-time employment	1481 (44.1)
	Part-time employment	313 (9.3)
	Student	71 (2.1)
	Retired	1153 (34.3)
	Unemployed	172 (5.1)
	Other	169 (5)

^a^MENA: Middle Eastern or North African.

^b^GED: General Educational Development.

### Descriptive Statistics of Engagement and Self-Reported Improvement Variables

Overall, users were highly engaged with the Elevate app as the average time since downloading was 1397 (SD 1281) days, which corresponds to 3.83 (SD 3.5) years. Users were active for an average of 120 (SD 130) weeks and an average of 5.42 (SD 1.42) days per week during active weeks and spent an average of 6.5 (SD 6.0) minutes per active day using the app (Table 3).

**Table 3:** Descriptive statistics of engagement variables.

Variable	Participants with data, n	Participants, mean (SD)
Time since downloading Elevate (d)	3367	1397 (1281)
Number of total active weeks	3361	120 (130)
Number of sessions per weeks	3361	5.42 (1.42)
Time spent per day (min)	3269	6.5 (6.0)

Most users reported improvement (ie, responding either “Better” or “Much better”) across all cognitive skills (range 1915/3365, 56.9%-2534/3366, 75.4%), most daily functioning items (range 1626/3361, 48.3%-2932/3364, 87.3%), and overall mental fitness (2480/3364, 84.4%). Reports of deterioration (ie, “Worse” or “Much worse”) were rare, ranging from 0.1% (3/3359) to 1.5% (53/3365) across all items (Table 4).

**Table 4:** Distribution of responses to self-report questions about Elevate experience.

Domain		Much worse, n (%)	Worse, n (%)	No change, n (%)	Better, n (%)	Much better, n (%)
**Cognitive skills**						
	Writing (n=3362)	1 (0)	18 (0.5)	1181 (35.1)	1652 (49.1)	510 (15.2)
	Speaking (n=3353)	2 (0.1)	26 (0.8)	1363 (40.7)	1538 (45.9)	424 (12.6)
	Reading (n=3358)	3 (0.1)	9 (0.3)	1275 (38)	1618 (48.2)	453 (13.5)
	Math (n=3366)	3 (0.1)	23 (0.7)	798 (23.8)	1741 (51.8)	793 (23.6)
	Memory (n=3365)	3 (0.1)	50 (1.5)	1397 (41.5)	1559 (46.3)	356 (10.6)
**Daily functioning**						
	Task efficiency (n=3360)	0 (0)	11 (0.3)	1398 (41.6)	1565 (46.6)	386 (11.5)
	Spending time intentionally (n=3363)	1 (0)	34 (1)	983 (29.2)	1740 (51.7)	605 (18)
	Keeping up with responsibilities (n=3364)	1 (0)	24 (0.7)	1713 (50.9)	1290 (38.3)	336 (10)
	Feeling motivated (n=3361)	0 (0)	3 (0.1)	476 (14.2)	1962 (58.4)	920 (27.4)
	Making personal progress (n=3362)	2 (0.1)	5 (0.1)	423 (12.6)	2019 (60.1)	913 (27.2)
**Overall mental fitness**						
	Mental fitness (n=3364)	0 (0)	5 (0.1)	519 (15.4)	2265 (67.3)	575 (17.1)

### Association Between Engagement Metrics and Improvements in Cognitive Skills

A series of ordinal logistic regressions was used to test the effects of the number of total active weeks, mean active days per week, and mean time per day on self-reported improvements in each of the cognitive skills, adjusting for age, gender, race, ethnicity, educational level, and time since downloading Elevate. After Bonferroni correction (α=.0015), a greater number of total active weeks was associated with a greater likelihood of reporting improvements in writing (OR 1.0027, 95% CI 1.0019-1.0036), speaking (OR 1.0022, 95% CI 1.0013-1.0030), reading (OR 1.0023, 95% CI 1.0014-1.0031), math (OR 1.0016, 95% CI 1.0007-1.0025), and memory (OR 1.0014, 95% CI 1.0006-1.0023). A greater number of mean active days per week was associated with a greater likelihood of reporting improvements in math (OR 1.15, 95% CI 1.09-1.22). Greater mean time per day on the app was associated with a greater likelihood of reporting improvements in speaking (OR 1.02, 95% CI 1.01-1.03), reading (OR 1.03, 95% CI 1.01-1.04), and math (OR 1.03, 95% CI 1.02-1.04; Table 5).

**Table 5:** Results from a series of ordinal logistic regressions conducted separately for each outcome (n=3160)a.

Outcome		Predictor										
		Number of active weeks				Mean active days per week				Mean time per day		
		*b* (SE)	OR^b^ (95% CI)	*P* value^c^	*b* (SE)		OR (95% CI)	*P* value^c^	*b* (SE)		OR (95% CI)	*P* value^c^
**Cognitive skills**												
	Writing	0.0027 (0.00045)	1.0027 (1.0019-1.0036)	<.001	−0.0011 (0.029)		1.00 (0.94-1.06)	.97	0.018 (0.0057)		1.02 (1.01-1.03)	.002
	Speaking	0.0022 (0.00044)	1.0022 (1.0013-1.0030)	<.001	0.026 (0.029)		1.03 (0.97-1.09)	.37	0.020 (0.0057)		1.02 (1.01-1.03)	<.001
	Reading	0.0023 (0.00044)	1.0023 (1.0014-1.0031)	<.001	0.050 (0.029)		1.05 (0.99-1.11)	.09	0.025 (0.0058)		1.03 (1.01-1.04)	<.001
	Math	0.0016 (0.00044)	1.0016 (1.0007-1.0025)	<.001	0.14 (0.029)		1.15 (1.09-1.22)	<.001	0.029 (0.0061)		1.03 (1.02-1.04)	<.001
	Memory	0.0014 (0.00044)	1.0014 (1.0006-1.0023)	.001	0.063 (0.029)		1.07 (1.01-1.13)	.03	0.017 (0.0056)		1.02 (1.01-1.03)	.002
**Daily functioning**												
	Task efficiency	0.0014 (0.00044)	1.0014 (1.0006-1.0023)	.001	0.055 (0.029)		1.06 (1.00-1.12)	.06	0.015 (0.0063)		1.02 (1.00-1.03)	.02
	Spending time intentionally	0.0016 (0.00044)	1.0016 (1.0007-1.0025)	<.001	−0.016 (0.029)		0.98 (0.93-1.04)	.58	0.017 (0.0063)		1.02 (1.00-1.03)	.008
	Keeping up with responsibilities	0.0016 (0.00044)	1.0016 (1.0007-1.0025)	<.001	0.063 (0.029)		1.06 (1.01-1.13)	.03	0.0079 (0.0064)		1.01 (1.00-1.02)	.22
	Feeling motivated	0.0017 (0.00046)	1.0017 (1.0008-1.0026)	<.001	−0.0088 (0.030)		0.99 (0.94-1.05)	.77	0.027 (0.0064)		1.03 (1.01-1.04)	<.001
	Making personal progress	0.0011 (0.00046)	1.0011 (1.0002-1.0020)	.02	0.093 (0.030)		1.10 (1.03-1.16)	.002	0.027 (0.0065)		1.03 (1.02-1.04)	<.001
**Overall mental fitness**												
	Mental fitness	0.0021 (0.00048)	1.0021 (1.0012-1.0031)	<.001	0.065 (0.032)		1.07 (1.00-1.14)	.04	0.042 (0.0066)		1.04 (1.03-1.06)	<.001

^a^All models adjusted for time since downloading the Elevate app, age, gender, race, ethnicity, and educational level.

^b^OR: odds ratio.

^c^The Bonferroni-corrected α on 33 tests was .0015.

### Association Between Engagement Metrics and Improvements in Daily Functioning and Mental Fitness

A series of ordinal logistic regressions was used to test the effects of the number of total active weeks, mean active days per week, and mean time per day on self-reported improvements in each of the daily functioning and overall mental fitness outcomes, adjusting for age, gender, race, ethnicity, educational level, and time since downloading Elevate. After Bonferroni correction (α=.0015), a greater number of active weeks was associated with a greater likelihood of reporting improvements in task efficiency (OR 1.0014, 95% CI 1.0006-1.0023), spending time intentionally (OR 1.0016, 95% CI 1.0007-1.0025), keeping up with responsibilities (OR 1.0016, 95% CI 1.0007-1.0025), feeling motivated (OR 1.0017, 95% CI 1.0008-1.0026), and overall mental fitness (OR 1.0021, 95% CI 1.0012-1.0031). Mean active days per week did not predict any self-reported change in daily functioning outcomes or overall mental fitness. Greater mean time per day on the app was associated with a greater likelihood of reporting improvements in feeling motivated (OR 1.03, 95% CI 1.01-1.04), making personal progress (OR 1.03, 95% CI 1.02-1.04), and overall mental fitness (OR 1.04, 95% CI 1.03-1.06; Table 5).

## Discussion

### Principal Findings

The purpose of this secondary analysis was to explore app engagement and self-reported cognitive benefits of Elevate, a commercial, personalized cognitive training app developed to support cognitive functioning. We aimed to (1) describe demographics, engagement metrics, and self-reported improvements; (2) examine associations between app engagement and self-reported improvements in cognitive functioning skills directly targeted by the app; and (3) examine associations between app engagement and self-reported improvements in daily functioning and overall mental fitness as potential transfer effects of cognitive training. Most Elevate users reported perceived improvements across cognitive skills, daily functioning, and overall mental fitness. Users who engaged with the app across more total active weeks (ie, used the app at least once during the week) were more likely to report improvements in all cognitive skill areas. More frequent use within active weeks (ie, using the app on more days) was linked to perceived improvement in math, whereas spending more time per day on the app was associated with improvements in speaking, reading, and math. Greater weekly engagement was also tied to perceived gains in aspects of daily functioning, including task efficiency, intentional time use, staying on top of responsibilities, and motivation. In addition, spending more time per day on the app was linked to feeling more motivated and making personal progress. Both more weeks of use and longer daily sessions were associated with a higher likelihood of users reporting improvements in overall mental fitness.

Elevate users were primarily middle-aged (mean age 55, SD 16 years), with most identifying as women (2184/3362, 64.96%) and White individuals (2557/3336, 76.65%), and most reported a high level of education (2274/3358, 67.72% had a college degree or higher). These findings align with those of previous research indicating that middle-aged, well-educated adults, especially women, are frequent users of digital health and cognitive platforms [38,39]. Engagement data indicated consistent use across multiple weeks, and most users endorsed perceived improvements in cognitive skills, daily functioning, and overall mental fitness (range: 1626/3361, 48.3%–2932/3364, 87.2%). These findings could be attributed to the self-selected sample, which may have excluded dissatisfied or less engaged users, or to placebo or expectancy effects. Given that middle age may be an important time for cognitive health behaviors, the engagement patterns observed in our sample suggest that this demographic may be receptive to cognitive training interventions, although additional research is needed to determine the app’s objective cognitive health benefits [40,41].

Elevate use was associated with perceived improvements in the core cognitive skills it was designed to target. More weeks of app use were associated with perceived improvements across all cognitive functioning domains, including writing, reading, speaking, math, and memory, which is in line with previous research showing that sustained engagement with cognitive training programs leads to improved cognitive functioning [42-44]. Engaging with the Elevate app on more days during an active week was associated with perceived improvement in math, whereas spending more time per day was linked to gains in speaking, reading, and math. These patterns may reflect differences in how cognitive skills are reinforced, with math potentially benefiting from frequent, distributed practice, whereas verbal skills may be more responsive to sustained, focused engagement that supports fluency and comprehension [45,46]. While previous studies of other commercial apps have reported gains in attention and processing speed [29,31], greater Elevate use was linked to self-reported improvements across a broad range of cognitive domains. Elevate’s personalized training structure, which tailors session content to individual skill levels and goals, may contribute to these patterns by aligning challenge and repetition to individual needs, thereby fostering engagement in targeted domains. While these associations were significant, the effect sizes were relatively small, limiting practical significance. Future research using validated outcome measures and objective cognitive assessments is critical to determine whether these small self-reported improvements reflect meaningful cognitive gains or are influenced by user expectations and recall bias.

A greater number of weeks with any app use was associated with perceived improvements in daily functioning, including task efficiency, intentional time use, keeping up with responsibilities, and motivation. In addition, users who spent more daily time on the app were more likely to report feeling motivated and making personal progress. Engaging in core cognitive skill training has been linked to self-regulation skills, such as planning, sustained effort, and goal-directed behavior, that relate to everyday productivity and well-being [47,48]. Such perceived transfer effects are rarely examined in commercial cognitive training studies, which tend to focus more narrowly on task-specific performance metrics [29,31,49,50]. While one study of a commercial cognitive training app showed that using a cognitive training app more often was linked to better scores on related cognitive tests [51], this study extends this work by examining the effects of cognitive training on user perceptions of functional improvements. However, the practical significance of these small effects remains uncertain as the mechanisms underlying users’ perceived benefits are unclear. Prior work in digital health has shown that psychological empowerment and enjoyment of app use can drive behavior change, pointing to potential mechanisms underlying users’ perceived functional gains in this study [52]. Future research should use validated functional outcome measures and experimental designs to test these mechanisms and determine which app features most effectively promote meaningful improvements in daily functioning.

To our knowledge, this is the first study to show that greater use of a commercial cognitive training app is linked to self-reported improvements in overall mental fitness. Much of the existing research on commercial cognitive training apps focuses on performance-based outcomes over the subjective abilities to regulate emotions, think clearly, and apply knowledge effectively (ie, capacities that define mental fitness [8-13,28,30,49,50]). This study builds on existing research by showing preliminary evidence that users may perceive psychological benefits from cognitive training. In doing so, it points to mental fitness as an outcome that merits scientific attention and the development of rigorous measurement tools. Future research should focus on creating and rigorously validating a standardized mental fitness assessment tool and also examine which aspects of Elevate’s training model (eg, domain variety, adaptive difficulty, and perceived personal relevance) are most effective in promoting sustained improvements in users’ mental fitness.

### Strengths and Limitations

This study offers several notable strengths. First, it linked self-reported outcomes with objective, real-world app use data from a commercial cognitive training platform, enabling a more ecologically valid understanding of how such tools are experienced in naturalistic settings. Second, it moves beyond traditional performance-based outcomes to assess a broader set of perceived benefits, including daily functioning and overall mental fitness. To our knowledge, this is one of the first studies to capture user perceptions of mental fitness, a rising area of interest that encompasses clarity of thought, emotional regulation, motivation, and the ability to apply cognitive skills in everyday life. Third, by analyzing detailed engagement metrics, this study identified use patterns associated with perceived gains, helping inform hypotheses about dose-response relationships. Fourth, this study examined a general population sample of adults—rather than clinical or older adult populations—offering insights into how cognitive training may benefit individuals in everyday, nonclinical contexts. Finally, this study contributes novel insights into user engagement and perceived cognitive and functional benefits of a widely available, commercial cognitive training app, leveraging data from a large, real-world sample to explore how digital tools may support cognitive health and mental fitness in everyday life.

There are also limitations. First, the cross-sectional study design without a control condition precludes us from determining whether our findings reflect genuine benefits of Elevate use, natural improvement over time, or user expectations about the app’s effectiveness. Future studies should use longitudinal experimental designs to better assess causal relationships between app engagement and outcomes. Second, this study relied entirely on self-reported outcomes, and the items used were developed specifically for this study rather than being drawn from validated instruments. Relatedly, the self-reported outcomes were based on the construct of mental fitness, which currently lacks an empirical definition and validated assessment tools. While we did assess perceived improvements in daily functioning domains and mental fitness relevant to everyday life, the absence of both objective outcome data and validated measurement tools limits the reliability and interpretability of these findings and could have led to measurement bias. Future research should incorporate validated cognitive and functional assessment tools, as well as behavioral or performance-based outcomes, to complement subjective reports. Fourth, this study did not include a control or comparison group, making it difficult to account for expectancy effects or alternative explanations for perceived change. Randomized controlled trials or matched comparison studies could help isolate the unique effects of the intervention. Fifth, the sample was predominantly composed of White, highly educated women, which limits the generalizability of the findings to more diverse populations. Finally, the self-selected sample was a relatively small proportion of the app’s total user base, which could have led to biased or inflated results because more engaged or satisfied users may have been more likely to volunteer to share their experiences in the survey. Future studies should include broader recruitment strategies to reach more diverse user profiles, including lower-engagement or disengaged users, and ensure representation across demographic groups.

### Conclusions

This study found that greater use of the Elevate app was associated with perceived improvements in a range of cognitive skills, including reading, writing, speaking, math, and memory, as well as various outcomes related to perceived daily functioning, including task efficiency, spending time intentionally, keeping up with responsibilities, feeling motivated, and making personal progress. Users also reported perceived gains in mental fitness, a multidimensional construct encompassing the ability to regulate emotions, think clearly, and apply knowledge effectively. Despite limitations such as unvalidated self-report measures and small effect sizes, these findings provide preliminary evidence that users see cognitive training apps as helpful not only for domain-specific skills but also for broader benefits. Future research using rigorous designs (eg, controlled trials) is needed to establish whether these perceptions reflect actual improvements in cognitive skills and daily functioning and determine which app features best support lasting cognitive and functional improvements.

## Appendix

Multimedia Appendix 1 Supplemental methods and results.

Multimedia Appendix 2 STROBE checklist for cross-sectional studies.

## Abbreviations

OR: odds ratio
STROBE: Strengthening the Reporting of Observational Studies in Epidemiology

## Data Availability

The datasets generated or analyzed during this study are not publicly available due to The Mind Company’s policy for user data but are available from the corresponding author on reasonable request.
