# Supplementary material for: User Engagement With and Perceived Impact of a Digital Cognitive Training App on Cognition, Daily Functioning, and Mental Fitness: Secondary Analysis of Cross-Sectional Survey Data
Source: JMIR Form Res. 2025 Oct 10;9:e80027. doi: 10.2196/80027 (PMC12552828; doi:10.2196/80027)
Supplement: Multimedia Appendix 1 [file formative_v9i1e80027_app1.docx]

User Engagement and Perceived Impact of a Digital Cognitive Training App on Cognition, Daily Functioning, and Mental Fitness: A Secondary Analysis of Cross-Sectional Survey Data from Elevate Users

**Supplemental Methods**

To test the proportional odds assumption, we compared the full proportional odds model (where the assumption was applied to all predictors) to a partial proportional odds model, in which the proportional odds constraint was selectively relaxed for each of the three predictors of interest ([citation](https://www.jstor.org/stable/2347760)). These partial proportional odds models allowed each of the three predictors to vary freely across outcome thresholds. We conducted likelihood ratio tests comparing proportional odds and partial proportional odds models for each predictor individually. A significant p-value in the likelihood ratio test (i.e., < .0015) was considered an indication of a violation of the proportional odds assumption (a Bonferroni correction for 33 tests was applied to the initial threshold of .05).

**Supplemental Results**

Results indicated that the vast majority of the models did not violate the proportional odds assumption for the predictors of interest. Results suggest that 3 coefficients violate the proportional odds assumption (Active Weeks predicting Memory, and Time Per Day predicting Task efficiency and Spending time intentionally), and one coefficient was not able to be tested ( Days per Week predicting Keeping up with Responsibilities). These coefficients should be interpreted with caution (Table S1).

| Table S1  Tests of the proportional odds assumption - results from likelihood ratio tests comparing proportional odds and partial proportional odds models for each predictor individually. A non-significant result indicates that the proportional odds assumption was not violated. | | | |
| --- | --- | --- | --- |
|  | Predictor - | | |
|  | Number of Active Weeks | Mean Active Days Per Week | Mean Time Per Day |
| Outcome | p* | p* | p* |
| *Cognitive Skills* | | | |
| Writing | .0049 | .080 | .75 |
| Speaking | .26 | .54 | .43 |
| Reading | .42 | .17 | .27 |
| Math | .70 | .063 | .14 |
| Memory | 0.00051 | .012 | .14 |
| *Daily Functioning* | | | |
| Task efficiency | 0.015 | .032 | 0.00010 |
| Spending time intentionally | 0.34 | .71 | 0.000022 |
| Keeping up with responsibilities | 0.09 | Invalid fit of the partial proportional odds model - not testable. | 0.0035 |
| Feeling motivated | 0.15 | .17 | .98 |
| Making personal progress | .91 | .48 | .98 |
| *Overall Mental Fitness* | | | |
| Mental fitness | .17 | .49 | .38 |
| *Bonferroni-corrected alpha on 33 tests is 0.0015.  Note: All models adjusted for time since downloading the Elevate app, age, gender, race, ethnicity, and education level. | | | |
